# Divergent roles of *ent*‐kaurene oxidase paralogs in rice momilactone biosynthesis

**DOI:** 10.1111/jipb.70262

**Published:** 2026-04-21

**Authors:** Zhibiao Wang, Yiling Feng, Leiming You, Meimei Xu, Kristin Helwig, Riqing Li, Si Nian Char, Bing Yang, Reuben J. Peters

**Affiliations:** ^1^ School of Life Sciences Beijing University of Chinese Medicine Beijing 100029 China; ^2^ Roy J. Carver Department of Biochemistry, Biophysics & Molecular Biology Iowa State University Ames 50011 IA USA; ^3^ Division of Plant Sciences, Bond Life Sciences Center University of Missouri Columbia 65211 MO USA; ^4^ Donald Danforth Plant Science Center St. Louis 63132 MO USA

**Keywords:** biosynthetic gene clusters, disease resistance, evolution

## Abstract

Rice (*Oryza sativa*) is a critically important food crop and a model cereal, with its momilactones among the first discovered phytoalexins. Notably, while momilactone biosynthesis is related to that of the gibberellin (GA) phytohormones, it was acquired via lateral gene transfer of an associated biosynthetic gene cluster (*mBGC*), but has been suggested to depend on paralog(s) of the *ent*‐kaurene oxidase (*KO*) required for GA production. Rice contains a five‐gene tandem array of the *KO* family, with three acting in GA biosynthesis (*OsKO1–3*), while the divergent KO‐like *OsKOL4* has been shown to catalyze reactions consistent with a role in the production of phytoalexins, such as the phytocassanes and momilactones. Here, building on biochemical characterization of distinct activity for *OsKOL5*, genetic evidence is provided indicating *OsKOL4* is much more important for momilactone biosynthesis, although both play a role in the production of the phytocassanes. Indeed, knock‐out lines (*kol4*, *kol5*, and *kol4/5*) are more susceptible, while overexpression increases resistance, to both the fungal blast pathogen *Magnaporthe oryzae* and bacterial blight pathogen *Xanthomonas oryzae*. Intriguingly, consistent with previously reported phytotoxicity of momilactone intermediates, a lesion mimic phenotype is observed with *kol4* and *kol4/5* but not *kol5* lines. Concordantly, phylogenomic analysis suggests acquisition of *mBGC* was preceded by tandem duplication of *KO* to generate *KOL4*, with subsequent duplication generating *KOL5*. Thus, despite the deleterious effects of *KOL4* loss, it can be speculated that linkage to the essential *KOs* may have alleviated such negative selection leading to *mBGC* recruitment, potentially relevant to plant *BGC* evolution more generally.

## Graphical abstract

Rice contains a five‐gene tandem array of genes encoding members of the *ent*‐kaurene oxidase (KO) family, with OsKOL4 and OsKOL5 playing divergent roles in momilactone metabolism, but both acting in the production of antifungal and antibacterial phytoalexins, which are important for disease resistance.

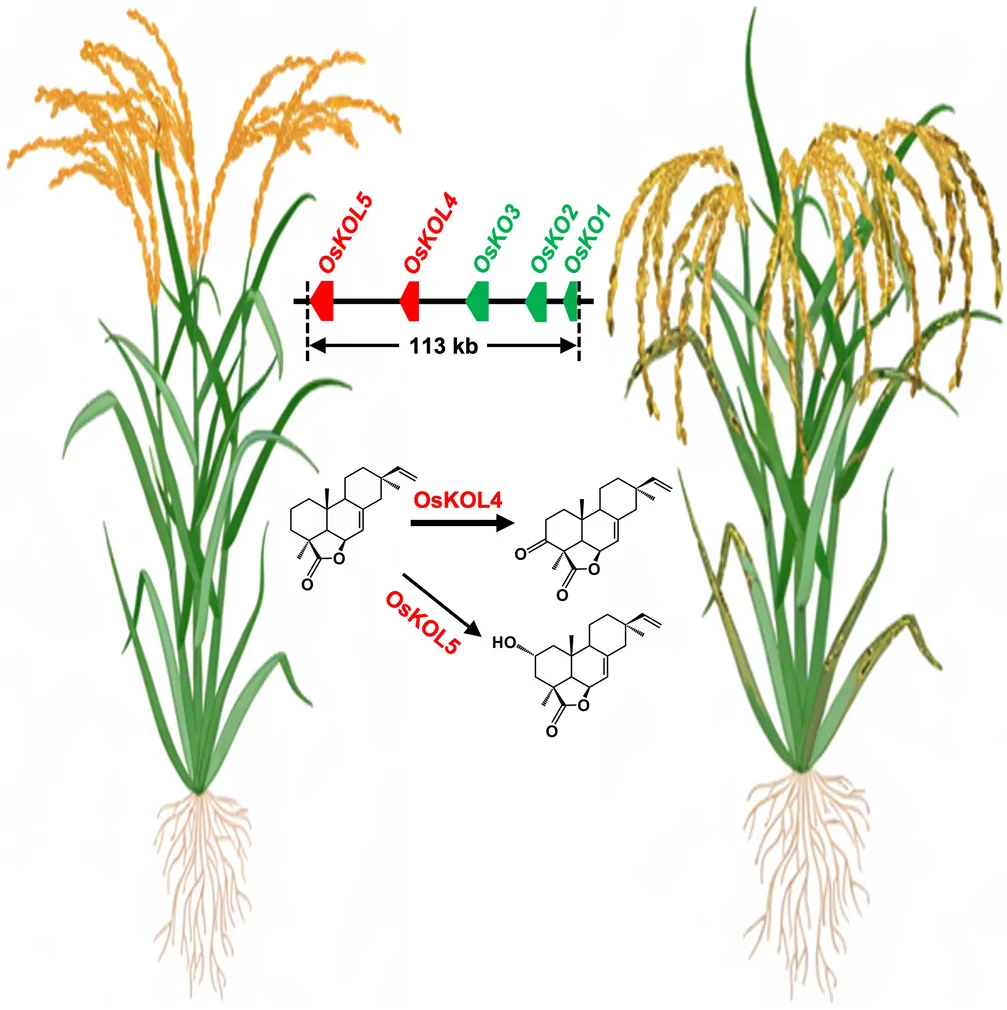

## INTRODUCTION

Rice (*Oryza sativa*), as a staple food grain, feeds two‐thirds of the world population (Sen et al., 2020). Critically, diseases can result in significant quality and yield losses in this important cereal crop. Among them, the most destructive are rice blast caused by the fungus *Magnaporthe oryzae*, with losses of up to 100%, with bacterial blight caused by the bacterium *Xanthomonas oryzae* pathovar (pv.) *oryzae* (*Xoo*), which leads to losses of 10%–50%, also prominent (Liu and Wang, 2016). During pathogen infection, plants produce an arsenal of antimicrobial natural products as part of their defense response, which are termed phytoalexins. The vast majority of rice phytoalexins are labdane‐related diterpenoids (LRDs), whose biosynthesis is derived from that of the gibberellin (GA) phytohormones (Peters, 2006), with the most prevalent being the momilactones and phytocassanes (Valletta et al., 2023). Indeed, the momilactones are among the first phytoalexins to have been identified (Cartwright et al., 1977).

LRDs are characterized by initial bicyclization of the general diterpenoid precursor (*E*,*E*,*E*)‐geranylgeranyl pyrophosphate (GGPP) via eponymous labdadienyl^+^ pyrophosphate intermediates in reactions catalyzed by class II diterpene cyclases (Peters, 2010). These enzymes most often produce copalyl pyrophosphate (CPP), such as the *ent*‐CPP stereoisomer relevant to GA biosynthesis and, hence, are termed CPP synthases (CPSs). Rice produces two CPP stereoisomers, with *ent*‐CPP formed by both *OsCPS1* and *OsCPS2*, and *syn*‐CPP by *OsCPS4* (Otomo et al., 2004; Prisic et al., 2004; Xu et al., 2004). Only *OsCPS1* is required for GA biosynthesis, while *OsCPS2* and *OsCPS4* are dedicated to more specialized LRD metabolism, dividing this into two distinct branches (Toyomasu et al., 2008).

**Figure 1:**
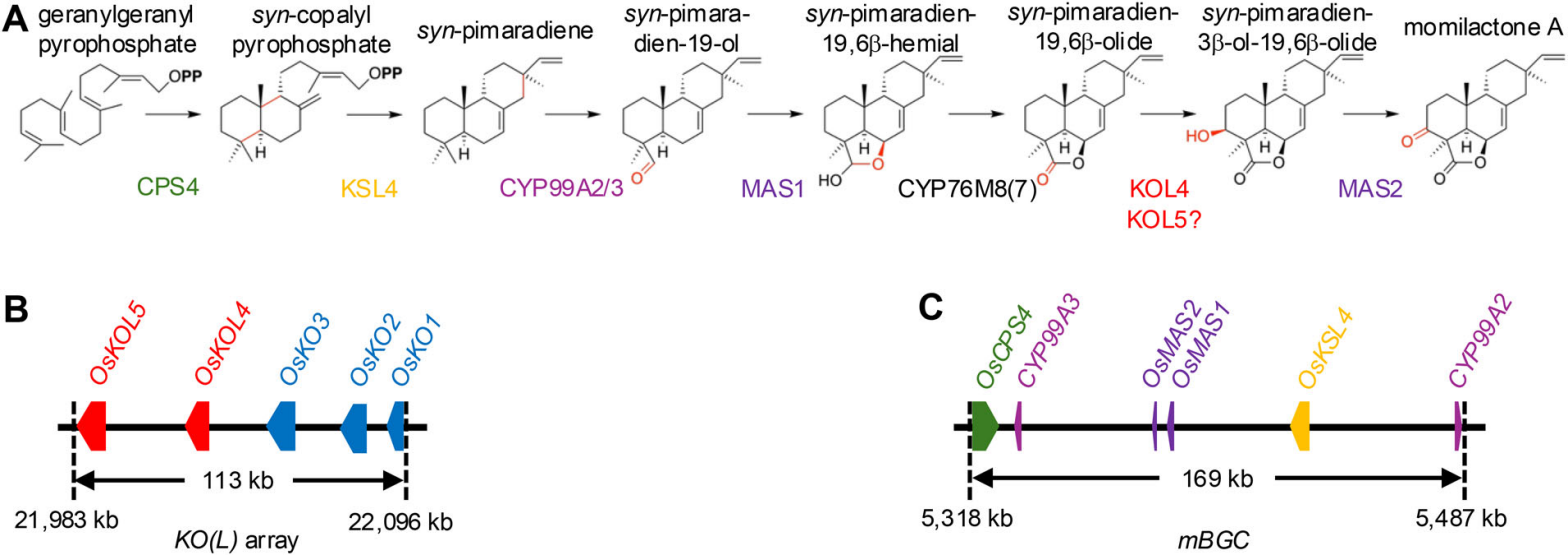
Rice momilactone biosynthesis and associated gene cluster, as well as *KO*(*L*) *tandem array* **(A)** Momilactone biosynthetic pathway, with *OsKOL4* highlighted by red text. **(B)** *KO(L)/CYP701A* tandem array on chromosome 6. Genes involved in gibberellin biosynthesis are shown in blue, with those involved in specialized metabolism shown in red. The arrows indicate the direction of transcription. Numbers below the diagram represent the physical locations (in kilobases) on chromosome 6. **(C)** Diagram of rice momilactone biosynthetic gene cluster (mBGC) on chromosome 4. All genes in the region are shown. The arrows indicate the direction of transcription. Colors indicate distinct enzymatic families. Numbers below the diagram represent the physical locations (in kilobases) on chromosome 4.

Biosynthesis of the various groups of rice LRDs is more specifically initiated by the subsequently acting class I diterpene synthases (Peters, 2006). That required for GA biosynthesis is the ubiquitous *ent*‐kaurene synthase (KS). In rice, this is encoded by *OsKS1* (Sakamoto et al., 2004). Given their phylogenetic relationship to KS, the others are termed KS‐like (KSLs), with rice containing an expanded family, *OsKSL2–14* (Xu et al., 2007; Miyamoto et al., 2016; Itoh et al., 2021; Feng et al., 2024). For example, OsKSL4 produces the *syn*‐pimaradiene precursor to the momilactones, and OsKSL7 produces the *ent*‐cassadiene precursor to the phytocassanes (Figure 1A).

In GA biosynthesis, the initially acting cytochrome P450 (CYP) is termed *ent*‐kaurene oxidase (KO). These are members of the CYP701A sub‐family and react with the carbon‐4(C4)α‐methyl (C19) to produce *ent*‐kaurenoic acid (Hedden, 2020). Consistent with their expanded *CPS* and *KS*(*L*) gene families, rice also contains an expanded family of *KO/CYP701A* sub‐family members encoded in a five‐gene tandem array (Figure 1B; Table S1). The semi‐dwarf rice mutant *d35* was found to correspond to *OsKO2/CYP701A6* (Itoh et al., 2004). Given that the introduction of *OsKO1/CYP701A7* also rescued the dwarf phenotype of the *d35* mutant, and knockout of *OsKO1* also showed dwarfism, clearly these two *KOs* are involved in GA biosynthesis (Itoh et al., 2004). In addition, it was later found that knocking out *OsKO3/CYP701A19* led to even more severe dwarfing than observed with *OsKO1/CYP701A7* knock‐outs, indicating *OsKO1*, *OsKO2*, and *OsKO3* all play roles in rice GA biosynthesis (Chen et al., 2019).

The other two CYP701A sub‐family members found in rice are phylogenetically distinct and, hence, are termed KO‐like (KOL), specifically *OsKOL4/CYP701A8* and *OsKOL5/CYP701A9* (Sakamoto et al., 2004). Notably, the introduction of *OsKOL4* did not rescue *d35* dwarfism, demonstrating that *OsKOL4* cannot be involved in GA biosynthesis (Itoh et al., 2004). Moreover, expression of *OsKOL4* and *OsKOL5* is induced by UV irradiation and/or chitin elicitation, suggesting roles in phytoalexin biosynthesis (Itoh et al., 2004). Recombinant *OsKOL4* was found to catalyze C3α‐hydroxylation of not only *ent*‐kaurene but also *ent*‐cassadiene, consistent with a role in phytocassane biosynthesis (Wang et al., 2012). Expression optimization revealed that *OsKOL4* can also catalyze C3β‐hydroxylation of *syn*‐pimaradiene (Kitaoka et al., 2015c), and was subsequently found to most likely mediate this transformation as a late step in momilactone biosynthesis (de la Pena and Sattely, 2021; Kitaoka et al., 2021).

Intriguingly, while it has been shown that rice contains a momilactone biosynthetic gene cluster (*mBGC*) (Figure 1C) (Shimura et al., 2007; Xu et al., 2012; Li et al., 2022), acquired by lateral gene transfer (Wu et al., 2022), it has also been shown that other *CYPs*, located in a separate, earlier evolving phytocassane *BGC* (*pBGC*) (Miyamoto et al., 2016), are required as well (Kitaoka et al., 2021). In addition, the composition of *pBGC* indicates that additional genes are required for the biosynthesis of the phytocassanes as well. Here, we examine the role of the *OsKOLs*, building on novel biochemical activity observed with *OsKOL5* and providing genetic evidence indicating that *OsKOL4* is much more important for momilactone biosynthesis, although both *OsKOL4* and *OsKOL5* play a role in the production of the phytocassanes and, thus, both are important in rice disease resistance. The lesion mimic phenotype observed with *kol4* knock‐out lines is consistent with previously suggested phytotoxicity of momilactone intermediates (Kitaoka et al., 2021), with phylogenomic analysis indicating earlier evolution of the *KOLs* relative to acquisition of the *mBGC*, providing insight into assembly of such *BGCs* in plants more generally.

## RESULTS

### Distinct activity for *OsKOL5* versus *OsKOL4* in momilactone biosynthesis

In previous work, using synthetic genes codon‐optimized for use in a bacterial (*Escherichia coli*) metabolic engineering system (Kitaoka et al., 2015a), no activity was detected with *OsKOL5* (Wang et al., 2012), even upon further improvement in the expression system (Kitaoka et al., 2015b). Given that yeast (*Saccharomyces cerevisiae*) has been successfully used as an expression platform for plant CYPs that could not be functionally expressed in *E. coli* (Pateraki et al., 2015), *OsKOL5* activity was investigated using this microbial host for recombinant expression. Based on its known activity, *OsKOL4* was utilized as a control. Accordingly, *OsKOL4* or *OsKOL5* were expressed with a CYP reductase from *Arabidopsis thaliana* (*AtR1*), and the resulting recombinant yeast strains were fed the momilactone precursor *syn*‐pimara‐7,15‐diene (Figure S1). Consistent with previously reported activity (Kitaoka et al., 2015b), *OsKOL4* was found to produce *syn*‐pimaradien‐3β‐ol. By contrast, *OsKOL5* transformed *syn*‐pimaradiene to a novel hydroxylated product. The distinct enzymatic activity exhibited by *OsKOL5* indicates functional divergence and a separate rather than redundant role for *OsKOL5* relative to *OsKOL4*, specifically in momilactone biosynthesis.

Given the role *OsKOL4* plays in catalyzing a late step in momilactone biosynthesis—that is, conversion of *syn*‐pimaradien‐19,6β‐olide to momilactone A (Kitaoka et al., 2021)—the ability of *OsKOL5* to react with this intermediate was tested. In particular, using feeding assays in engineered yeast strains, again with *OsKOL4* as a control. Similarly consistent with previous results (Kitaoka et al., 2021), *OsKOL4* efficiently converted *syn*‐pimaradien‐19,6β‐olide to the C3‐keto derivative momilactone A (Figure 2A, B). While *OsKOL5* exhibited lower catalytic efficiency, it produced a novel hydroxylated product (Figure 2C, D).

**Figure 2:**
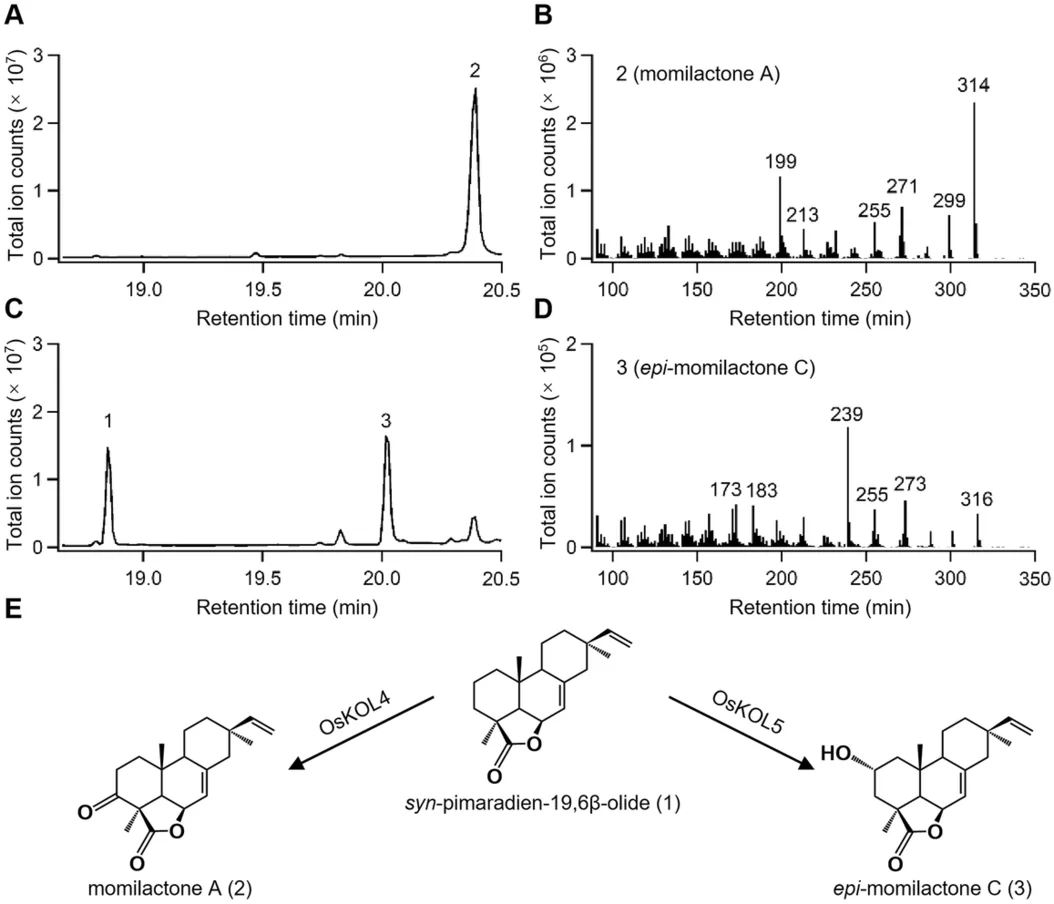
Catalytic activity of *OsKOL4* and *OsKOL5* **(A)** GC–MS chromatogram from a feeding assay of *OsKOL4* with *syn*‐pimaradien‐19,6β‐olide (1) showing the production of momilactone A (2). **(B)** Mass spectrum of momilactone A (2). **(C)** GC–MS chromatogram from a feeding assay of *OsKOL5* with *syn*‐pimaradien‐19,6β‐olide (1) showing the production of a novel product, which was determined to be *epi*‐momilactone C (3). **(D)** Mass spectrum of *epi*‐momilactone C (3). **(E)** Reactions catalyzed by *OsKOL4* and *OsKOL5*. *OsKOL4* catalyzes 3β hydroxylation of *syn*‐pimaradien‐19,6β‐olide and subsequent oxidation to the 3‐keto containing momilactone A. By contrast, *OsKOL5* catalyzes 2α hydroxylation, producing *epi*‐momilactone C, whose chemical structure was determined by NMR.

To produce enough of the *OsKOL5* product for structural characterization by Nuclear Magnetic Resonance (NMR), small‐scale fed cultures were combined (totaling ~3 L) for the extraction of this compound. After purification by flash chromatography and high‐performance liquid chromatography (HPLC), ~1 mg of product was obtained. From the subsequent NMR structural analysis (Figures S2–S4; Table S2), this derivative was determined to be 2α‐hydroxy‐*syn*‐pimaradien‐19,6β‐olide (Figure 2E). To distinguish this from the previously reported 2β‐hydroxylated momilactone C (Mitsuaki et al., 1976), the *OsKOL5* product was termed *epi*‐momilactone C.

### Only *OsKOL4* is important in momilactone biosynthesis

While previous biochemical analysis has demonstrated that *OsKOL4* has 3‐hydroxylase activity relevant to both phytocassane and momilactone biosynthesis (Kitaoka et al., 2015b), the activity observed here with *OsKOL5* seems distinct. Nevertheless, redundancy between these remains possible. To investigate this, both *OsKOL4* (OsKitaake06g191200) and *OsKOL5* (OsKitaake06g191000), were targeted using a previously described CRISPR/Cas9–2gRNA genome editing system to create knock‐outs in the model subspecies (ssp.) japonica cultivar (cv.) Kitaake (Bi and Yang, 2017; Char et al., 2019). For each gene, three different mutant lines containing distinct premature stop codons were isolated by genotyping in the T1 generation (Figure S5), and selfed to generate CRISPR construct‐free homozygous lines, *kol4* and *kol5*, respectively. In addition, a multiplex CRISPR/Cas9 approach was used to knock out both *KOLs* simultaneously. Specifically, *kol4/5* double knock‐out lines were created by targeting sequences that are identical in both genes (Figure S6), again with three distinct missense and CRISPR‐construct free lines generated as above.

To further investigate their activity, *OsKOL4* and *OsKOL5* were individually overexpressed under the control of the 35S promoter. Analysis by RT‐PCR enabled selection of three transgenic lines for each, with dramatic increases in mRNA levels of the relevant gene compared to the parental/wild‐type (WT) cv. Kitaake (Figure S7). Notably, *KOL4‐OE* transgenic plants exhibited somewhat impaired growth, reaching only 85%–89% of the height of WT cv. Kitaake at the mature stage. By contrast, *KOL5‐OE* plants and all the CRISPR knockout lines (*kol4*, *kol5*, *kol4/5*) did not exhibit plant height‐related phenotypes (Figure S8). Based on the previously observed 3α‐hydroxylation of *ent*‐kaurene by *OsKOL4* (Wang et al., 2012), it seems likely that overexpression of *OsKOL4* leads to diversion of this precursor away from GA biosynthesis.

The effects of these manipulations of *OsKOL4* and/or *OsKOL5* on the prevalent momilactones A & B and phytocassanes A–F were determined via a previously developed targeted LC–MS/MS method for analysis of extracts from induced leaves (Lu et al., 2018; Zhang et al., 2021). Consistent with the previously hypothesized role for *OsKOL4* in momilactone biosynthesis, relative to WT cv. Kitaake accumulation of momilactone A is significantly reduced in the *kol4* and *kol4/5* lines (Figure 3A), although levels of the derived but less prevalent momilactone B were only moderately reduced (Figure S9A). By contrast, the *kol5* lines did not exhibit such decreases (Figures 3A, S9A). Similarly, the *KOL4‐OE* but not *KOL5‐OE* lines exhibited significant increases relative to WT plants in the production of momilactone A (Figure 3A), as well as momilactone B (Figure S9B). Consistent with the recombinant activity observed here, chemotypic analysis found that *epi*‐momilactone C levels, already quite low, were further reduced relative to WT in *kol5* and *kol4/5* but not in *kol4*. However, these levels were not significantly increased in *KOL5*‐OE (nor *KOL4‐OE*) lines (Figure S10), indicating that this activity is only incidental.

**Figure 3:**
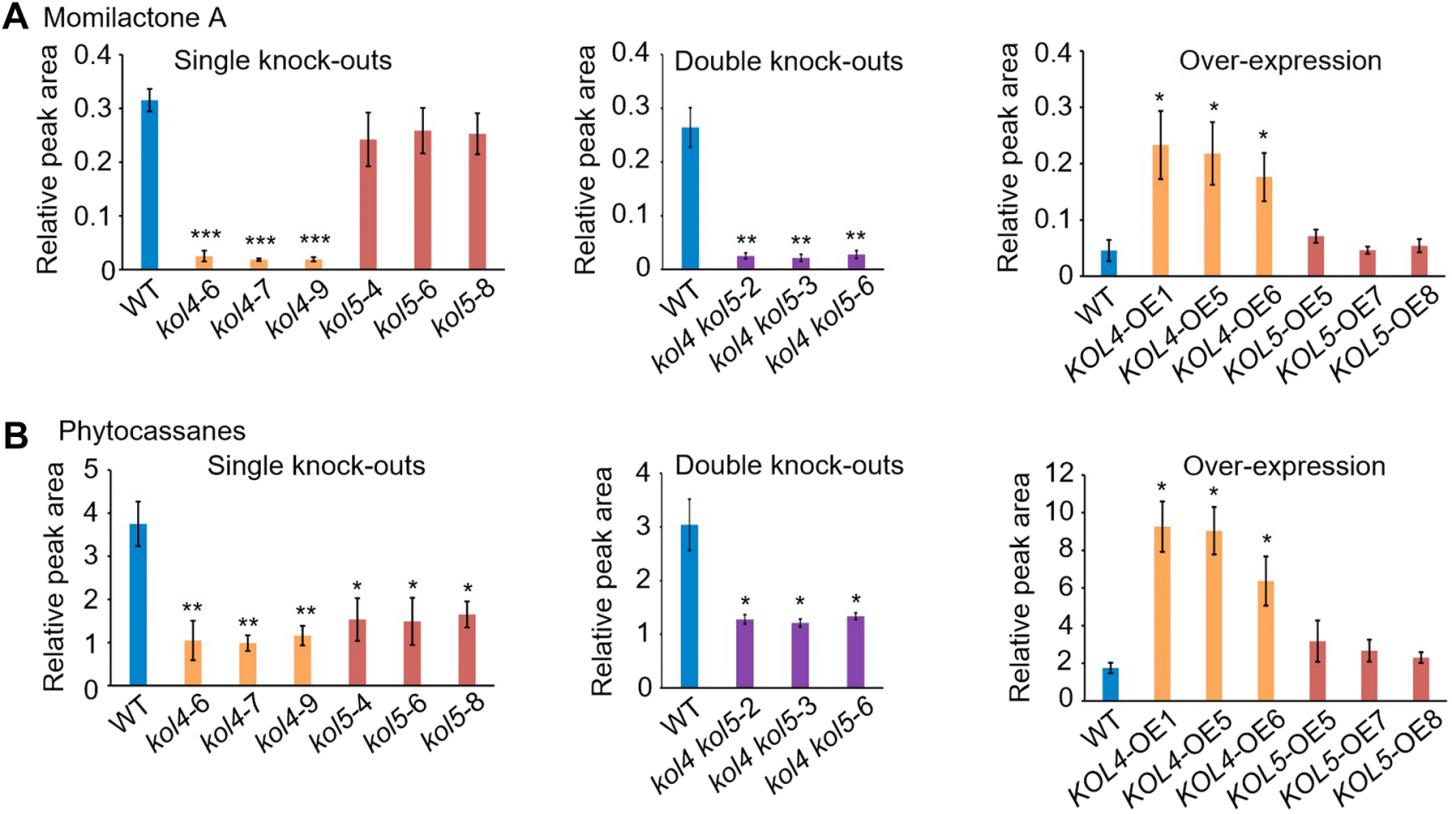
**Effects of genetic manipulation of** ****OsKOL4**** **and** ****OsKOL5**** **on momilactone and phytocassane production** **(A**, **B)** Effect of genetic manipulation of *OsKOL4* and/or *OsKOL5* on targeted labdane‐related diterpenoid production in induced leaf tissues. Effects on momilactone A levels of individually knocking‐out *OsKOL4* or *OsKOL5* (as indicated; left), or both (double knock‐out; middle), or overexpression of *OsKOL4* or *OsKOL5* (as indicated; right) **(A)**. Effects on aggregated phytocassane levels of individually knocking‐out *OsKOL4* or *OsKOL5* (as indicated; left), or both genes (middle), or overexpression of these genes (as indicated; left) **(B)**. Values are given as means ± *SE* (*P < 0.05; **P < 0.01; ***P < 0.001 compared with wild‐type using Student's *t*‐test).

Intriguingly, decreased accumulation of phytocassanes was observed in both *kol4* and (to a somewhat lesser extent) *kol5*, as well as *kol4/5* lines relative to WT (Figure 3). However, while *KOL4‐OE* lines exhibited significant increases relative to WT plants in the production of phytocassanes, no dramatic increases in the accumulation of either momilactones or phytocassanes were observed in *KOL5‐OE* plants (Figures 3A, B, S9B). Thus, while *OsKOL4* is important for momilactone and phytocassane biosynthesis, *OsKOL5* exhibits a separate role from *OsKOL4* in phytocassane biosynthesis, although the lack of effect upon overexpression suggests that its activity is not rate‐limiting and there is no appreciable difference in amounts of the differentially oxygenated distinct phytocassanes, leaving any differences between the roles of the two KOLs in such biosynthesis opaque.

### Both KOLs are important for defense

The increased susceptibility resulting from knocking‐out the upstream *OsCPS2* indicates that phytocassanes serve as effective phytoalexins against the rice fungal blast pathogen *M. oryzae* and bacterial pathogen *X. oryzae* (Lu et al., 2018; Zhang et al., 2021). Given the effect that manipulation of *OsKOL4* and *OsKOL5* had on phytocassane accumulation, the susceptibility of these lines was investigated here. Fungal blast was assayed by spray‐inoculating rice seedlings at the three‐leaf stage with *M. oryzae* and analyzing the diseased (discolored) leaf area. Notably, *kol4* and *kol4/5* plants were clearly more susceptible to *M. oryzae* than WT, while *kol5* exhibited a smaller but still significant increase in susceptibility as well (Figure 4A, B), approximating their relative effect on phytocassane levels. By contrast, despite their differing effect on the production of these phytoalexins, both *OsKOL4‐OE* and *OsKOL5‐OE* lines exhibited significantly smaller infected areas than WT (Figure 4C, D).

**Figure 4:**
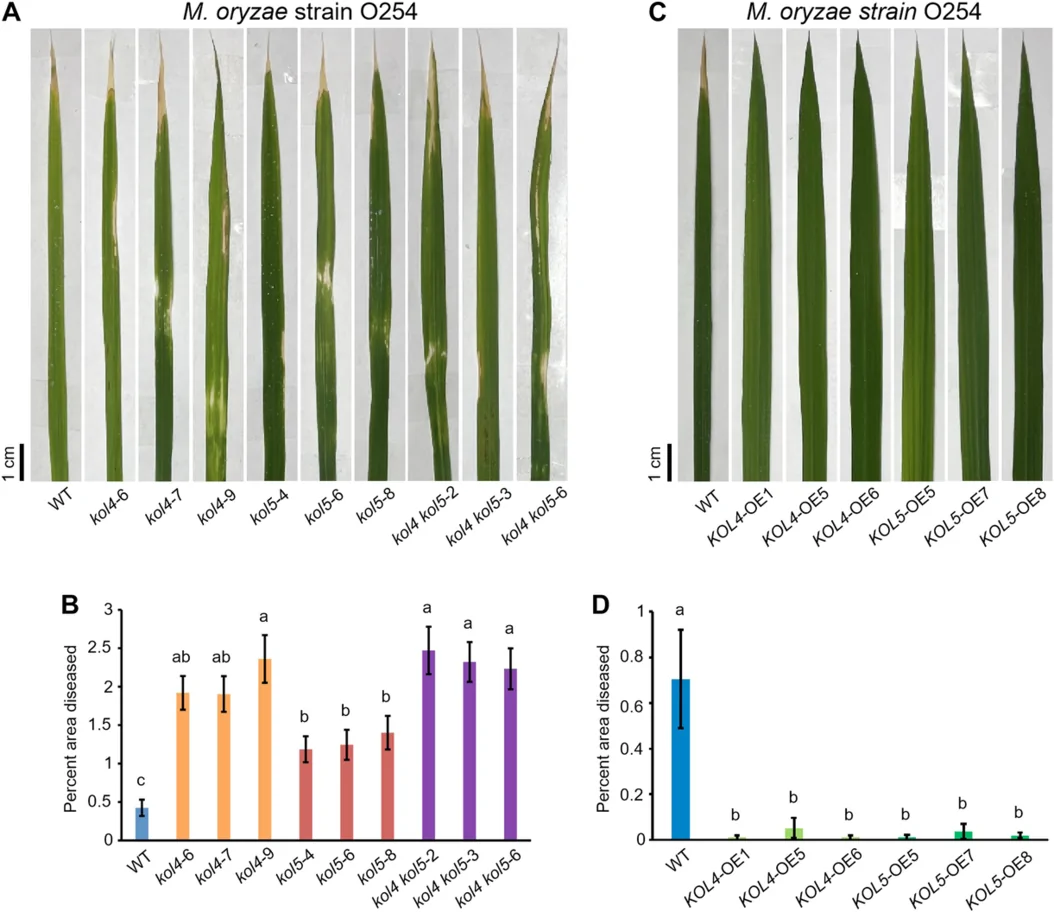
**Effect of genetic manipulation of** ****OsKOL4**** **and** ****OsKOL5**** **on susceptibility to** ***Magnaporthe oryzae*** **(A)** *OsKOL4* and *OsKOL5* knock‐out mutants exhibit increased susceptibility to *M. oryzae*. Leaves from 3‐week‐old WT, *kol4*, *kol5*, and *kol4/5* plants after infection with *M. oryzae* strain O254 by spraying spore suspension. **(B)** Percentage of diseased (discolored) area versus total leaf area for WT, *kol4*, *kol5*, and *kol4/5* lines 6 d post‐infection with *M. oryzae* strain O254. **(C)** *OsKOL4* and *OsKOL5* overexpression lines exhibit enhanced resistance to *M. oryzae*. Leaves from 3‐week‐old WT, *KOL4‐OE*, and *KOL5‐OE* plants after infection with *M. oryzae* strain O254 by spraying spore suspension. **(D)** Percentage of diseased (discolored) area versus total leaf area for WT, *KOL4‐OE*, and *KOL5‐OE* lines 6 d post‐infection with *M. oryzae* strain O254. Values in **(C**, **D)** are given as means ± *SE*. Significant differences are indicated by different letters (*P* < 0.05, two‐sided Fisher's least significant difference).

Bacterial blast was assayed by infiltration of 3‐week‐old seedlings with *Xoo*, and bacterial numbers were measured 2 d post‐inoculation. Again, consistent with their effect on phytocassane levels, *kol4*, *kol5*, and *kol4/5* plants were all more susceptible to *Xoo* relative to WT, with up to twofold increases in resulting colony‐forming units (CFU) (Figure 5A, B). However, despite their differing effects on phytocassane levels, both *KOL4‐OE* and *KOL5‐OE* exhibited significantly increased resistance relative to the WT line, with up to twofold decreases in CFUs (Figure 5C, D). The related bacterial leaf streak pathogen *X. oryzae* pv. *oryzicola* (*Xoc*) was also assayed. Similarly, *kol4*, *kol5*, and *kol4/5* mutants were found to also exhibit increased susceptibility to *Xoc* relative to WT, with similar up to twofold increases in resulting CFUs, while *KOL4‐OE* and *KOL5‐OE* showed enhanced resistance (Figure S11).

**Figure 5:**
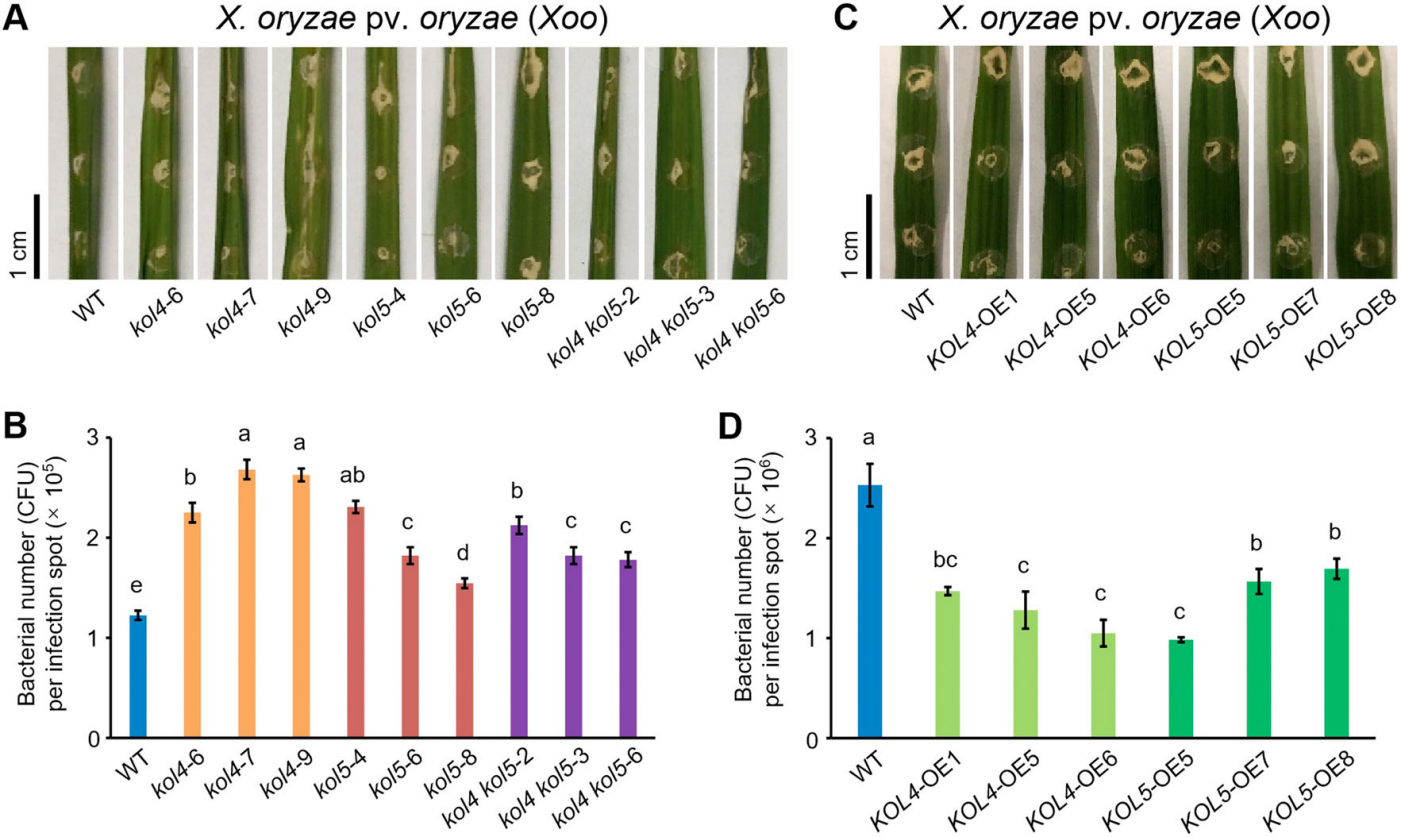
Effect of genetic manipulation of *OsKOL4* and *OsKOL5* on susceptibility to *Xanthomonas oryzae* pv. *oryzae* (*Xoo*) **(A)** *OsKOL4* and *OsKOL5* knock‐out mutants exhibit increased susceptibility to *Xoo*. Leaves from 3‐week‐old WT, *kol4*, *kol5*, and *kol4/5* plants after inoculation with *Xoo* strain PXO99^A^ via infiltration with a needleless syringe. **(B)** Bacterial number per inoculation spot of WT, *kol4*, *kol5*, and *kol4/5* plants 3 d post‐infection with *Xoo* strain PXO99^A^. **(C)** *OsKOL4* and *OsKOL5* overexpression lines exhibit enhanced resistance to *Xoo*. Leaves from 3‐week‐old plants of WT, *KOL4‐OE*, and *KOL5‐OE* lines after inoculation with *Xoo* strain PXO99^A^ via infiltration with a needleless syringe. **(D)** Bacterial number per inoculation spot of WT, *KOL4‐OE*, and *KOL5‐OE* lines 3 d post‐infection with *Xoo* strain PXO99^A^. Values in **(C**, **D)** are given as means ± *SE*. Significant differences are indicated by different letters (*P* < 0.05, two‐sided Fisher's least significant difference).

Taken together, these results indicate that both *OsKOL4* and *OsKOL5* play a positive role in rice disease resistance against both the fungal pathogen *M. oryzae* and the bacterial pathogen *X. oryzae*. However, while the susceptibility of the knock‐out lines is correlated with their effects on phytocassane levels, as is the increased resistance observed with *KOL4‐OE*, that of *KOL5‐OE* indicates that such overexpression stimulates production of other phytoalexins.

### *OsKOL4* knock‐out leads to a lesion mimic phenotype

Strikingly, knocking out *OsKOL4* results in a lesion mimic phenotype. For example, the representative *kol4‐7* line displayed a mild to severe lesion mimic phenotype, as does the representative *kol4/5‐3* double knock‐out line, while the representative *kol5‐6* line does not (Figure S12). This matches the lesion mimic phenotype also reported with mutant lines in which the *pBGC* was deleted, and which can be suppressed by deletion of the *mBGC* associated with momilactone biosynthesis (located on chromosome 4 and also termed *c4BGC*, with the deletion mutant designated *Δc4bgc*). The phenotype was more specifically assigned to the *CYP76M7* and *CYP76M8* contained within the *pBGC*, which exhibit partial redundancy for momilactone biosynthesis, supporting the accumulation of phytotoxic intermediates (or their derivatives) from blocking momilactone production (Li et al., 2022). To investigate if the same applies to *OsKOL4*, given that it also plays a role in momilactone biosynthesis, this was knocked out in the *Δc4bgc* line to create *Δc4bgc/kol4* double mutant lines (Figure S13). Supporting the hypothesis that it is the loss of *OsKOL4* activity in momilactone biosynthesis that leads to the lesion mimic phenotype, under the same conditions where this is observed in *kol4* lines, it is not in *Δc4bgc/kol4* lines (Figure 6).

**Figure 6:**
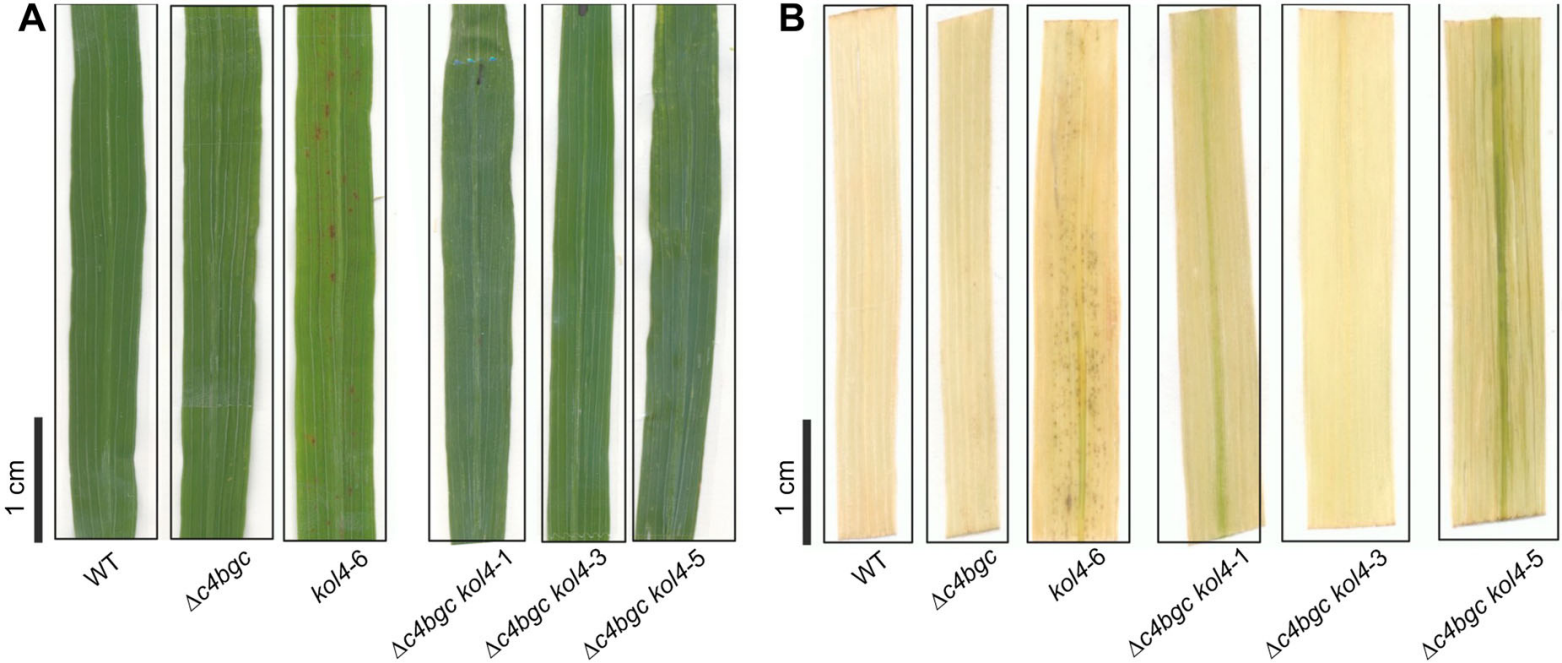
**The lesion mimic phenotype is associated with the role of** ***KOL4*** **in momilactone biosynthesis** **(A)** The lesion mimic phenotype in *kol4* is suppressed by the loss of the momilactone‐associated biosynthetic gene cluster on chromosome 4 (*Δc4bgc*), as shown with representative leaves from flowering stage plants grown at 30°C in a gree nhouse. **(B)** 3,3′‐diaminobenzidine (DAB) staining of leaves after the onset of the lesion‐mimic phenotype reveals hydrogen peroxide accumulation, indicating a role for reactive oxygen species in this necrosis.

### Evolution of the *KO(L)/CYP701A* gene family in *Oryza*

Reflecting their role in requisite phytohormone biosynthesis, *KO/CYP701A* sub‐family members are found in all plant species. As previously noted (Ding et al., 2019), upon phylogenetic analysis, the dicot and monocot KOs form distinct clades, with the rice KOs and KOLs forming deeply divided separate groups even within the monocot clade. This suggests that the rice *KOL* gene(s) arose from an early tandem duplication and underwent neofunctionalization under positive selection pressure for diversion from GA biosynthesis to more specialized (phytoalexin) metabolism.

The locus corresponding to the *O. sativa KO*(*L*) five‐gene tandem array is conserved in six closely related *Oryza* species, which all contain AA‐type genomes, specifically *Oryza rufipogon*, *Oryza nivara*, *Oryza barthii*, *Oryza glaberrima*, *Oryza glumaepatula*, and *Oryza meridionalis* (Figure S14). To further investigate the evolutionary origins of the rice *KOLs*, six diploids (*n* = 12) of distinct genome types, specifically AA (*O. sativa*), BB (*Oryza punctata*), CC (*Oryza officinalis*), EE (*Oryza australiensis*), FF (*Oryza brachyantha*), and GG (*Oryza meyeriana*), as well as a tetraploid (*n* = 24) KKLL (*Oryza coarctata*), were subjected to phylogenomic analysis using *Leersia perrieri* as outgroup (Figure 7A). Phylogenetic and syntenic analysis indicate that, while all contain both *KO* and *KOL* homologs at the same locus, there is some variation between these species (Figure 7B, C). Specifically, *O. punctata* (BB) and *O. brachyantha* (FF) contain two *KOL* paralogs and one *KO* (*OpKOL‐a*, *‐b* & *OpKO* and *ObKOL‐a*, *‐b* & *ObKO*), while *O. officinalis* (CC), *O. australiensis* (EE), *O. meyeriana* (GG), and both *O. coarctata* sub‐genomes (KK & LL) contain single *KO* and *KOL* homologs. In fact, even the *L. perrieri* genome contains a homoeologous region, containing one *KO* and two *KOL* genes (*LpKOL‐a*, *‐b*). Thus, it seems likely that *KO* and *KOL* diverged before the *Leersia/Oryza* ancestor, and both have been subject to gene duplication within *Oryza*.

**Figure 7:**
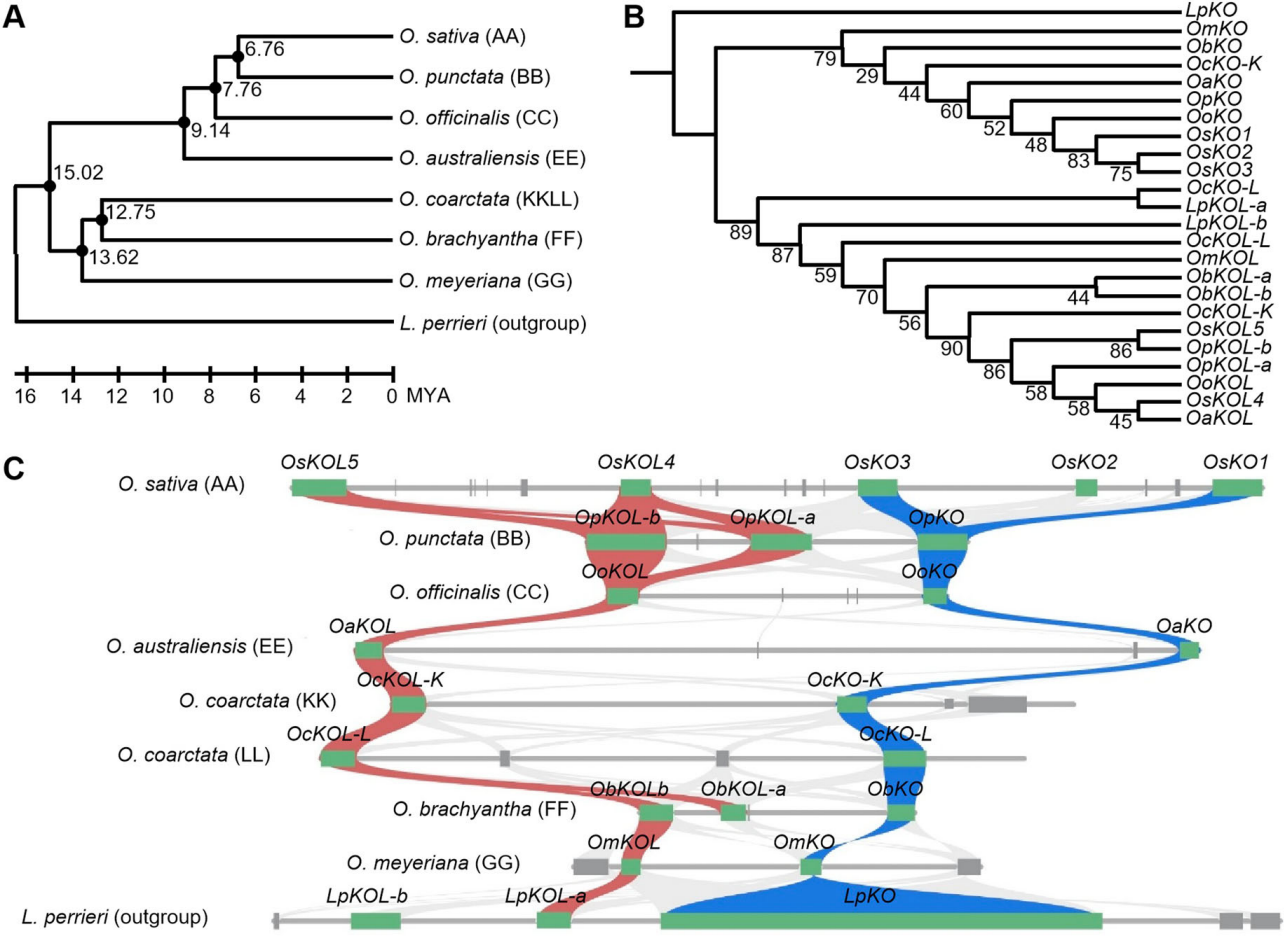
Phylogenetic and syntenic analyses of KO(L) homologs in Oryzoideae **(A)** Time‐dated phylogenetic tree of seven wild species of *Oryza* and *Leersia*. The maximum likelihood phylogeny was constructed with IQ‐TREE using 310 single‐copy genes. Divergence time was obtained using the molecular calibration for the crown age of *Oryza* as 15 MYA (million years ago). **(B)** Phylogenetic tree of KO(L) homologs in seven wild species of *Oryza* and *Leersia*. The tree was built with IQ‐TREE using the maximum likelihood method with 1,000 bootstrap replicates. **(C)** Syntenic analysis of the KO gene tandem array in seven wild species of *Oryza* and *Leersia*. The blue and red ribbons show the syntenic relationships between KO and KOL, respectively.

Within the *Oryza* AA genome lineage, both *KOL4* and *KOL5* are uniformly present, with orthologs of both present throughout the fully sequenced species available from the Gramene *Oryza* database (Tello‐Ruiz et al., 2022)—that is, *O. rufipogon*, *O. nivara*, *O. barthii*, *O. glaberrima*, *O. glumaepatula*, and *O. meridionalis* (Stein et al., 2018). Moreover, within a phylogenetically representative set of *O. sativa* cultivars also present in the Gramene *Oryza* database (Zhou et al., 2020), the orthologs of each share coding sequence identities of > 99.4% for *OsKOL4* and > 97.8% for *OsKOL5*. Indeed, even within the large dataset (> 4,700 accessions) utilized by RiceVarMap2 (Zhao et al., 2021), *OsKOL4* contains no “damaging” single‐nucleotide polymorphism (SNP) or insertion/deletion (indel), and in *OsKOL5* only one “damaging” SNP and one indel are present, and both occur at < 2% frequency (Figure S15). The observed high conservation levels indicate strong positive selection pressure for retention of *KOL* activity in *Oryza*.

## DISCUSSION

Consistent with the previously reported phylogenetic divergence and inducible transcription (Itoh et al., 2004), the rice *OsKOL4* and *OsKOL5* are shown here to no longer operate in GA biosynthesis but rather in more specialized LRD metabolism. Neither catalyzes the conversion of *ent*‐kaurene to *ent*‐kaurenoic acid relevant to GA production. Instead, upon recombinant expression in yeast, *OsKOL5* was found to exhibit distinct catalytic activity in momilactone biosynthesis compared with *OsKOL4*. Specifically, with *syn*‐pimaradiene *OsKOL5* hydroxylates at a different position than *OsKOL4* and, more importantly, also does so with the momilactone intermediate *syn*‐pimaradien‐19,6β‐olide, producing the C2α‐hydroxyl derivative termed here *epi*‐momilactone C rather than the C3‐keto derivative momilactone A produced by *OsKOL4* (Figure 2). Moreover, the genetic evidence further supports the separation of these from GA biosynthesis, as knock‐out lines did not exhibit a full dwarf phenotype. Although overexpression of *OsKOL4* did lead to semi‐dwarfed plants, this is almost certainly derived from the ability to catalyze divergent 3α‐hydroxylation of *ent*‐kaurene, directing this precursor away from GA biosynthesis.

Further consistent with the observed biochemical activity, the genetic evidence indicates distinct activity for *OsKOL4* and *OsKOL5* in momilactone biosynthesis. More specifically, that *OsKOL4* plays a crucial role in the pathway to the major products momilactones A & B, while *OsKOL5* serves a minor divergent role for production of the trace product *epi*‐momilactone C. The importance of *OsKOL4* for momilactone biosynthesis is further supported by the lesion‐mimic phenotype observed in *kol4* (and *kol4/5*) plants, which resembles previously reported downstream disruption of this pathway and has been attributed to phytotoxicity of intermediate metabolites. Conversely, both *OsKOL4* and *OsKOL5* appear to be important for phytocassane biosynthesis, albeit *OsKOL4* seems to play a somewhat greater role at least upon overexpression, consistent with the increased susceptibility of *kol4* (and *kol4/5*) plants to at least *M. oryzae*.

Phylogenomic analysis reveals duplication from *KO* and neofunctionalization to *KOL* occurred before divergence of the Oryzoideae, with further duplication of *KOL* arising in parallel several times within this plant sub‐family. Intriguingly, it has been reported that the *mBGC* was acquired by lateral gene transfer within the Oryzoideae, specifically the last common ancestor of the *Oryza* genus (Wu et al., 2022). This then followed the evolutionary appearance of *KOL*, as shown here (Figure 7C). Notably, once established in the population, linkage of *KOL* to the requisite *KO* in the observed gene array, despite the deleterious lesion mimic phenotype observed upon knocking out *OsKOL4*, would alleviate such negative selective pressure. Such establishment of the ancestral *KOL* can be attributed to its role in the biosynthesis of more widespread and similarly earlier evolved phytocassanes (Miyamoto et al., 2016). Given the importance of these LRD phytoalexins in rice plant defense against both fungal and bacterial pathogens (Lu et al., 2018; Zhang et al., 2021), this presumably provided the strong driving force for evolution and retention of *KOLs* in the Oryzoideae observed here. In addition, the importance of both *OsKOL4* and *OsKOL5* in rice phytocassane phytoalexin biosynthesis provides the selective pressure for their dual retention in the *Oryza* genus, also observed here.

In conclusion, the results reported here provide not only insight into the evolution of the *KO*(*L*)/CYP701A sub‐family, but also the evolution of LRD biosynthesis in rice, as well as that of BGCs more generally. Specifically, it can be hypothesized that the original derivation of the *KOL*(*s*) was driven by the need to produce effective LRD phytoalexins, such as the phytocassanes, biosynthesis of which then provides the selective pressure for their retention. Prior evolution of a *KOL*, presumably able to contribute to momilactone biosynthesis, then enabled acquisition of the associated incomplete *mBGC*. Moreover, it can be further hypothesized that, despite the negative pressure against loss of this *KOL* exemplified by the lesion mimic phenotype, its linkage to the *KO*(*s*) required for GA biosynthesis may have alleviated such selection for recruitment to the *mBGC*. Strikingly, this provides novel evidence for previous speculation that such linkage of genes involved in more specialized metabolism to an essential gene serves as a rationale for the observation of incomplete BGCs in plants more generally (Smit and Lichman, 2022).

## MATERIALS AND METHODS

### Plant materials and growth conditions

All knock‐out and overexpression plants used in this study were in the cv. Kitaake background. For germination, seeds were surface‐sterilized with 70% ethanol for 1 min and 20% (v/v) bleach for 15 min, washed with water four times, and dispersed on half‐strength Murashige and Skoog (MS) medium with 0.8% agar (pH = 5.7). These seedlings were grown at 30°C for 10 d, then transferred to pots filled with farm soil, and planted in the growth chamber under a photoperiod of 12 h light at 30°C and 12 h dark at 28°C.

### Yeast constructs and feeding assays

Construction of yeast expression vectors was carried out via the in‐fusion cloning system. Full‐length coding sequences for *KOL4* and *KOL5* were amplified from a rice cDNA library with specific primers containing overhangs for further cloning (all primers used here can be found in Table S3). Similarly, the backbone of the destination vector *pAGT3301* (Bathe et al., 2019), including *GAL* promoter, *URA3* gene, and *AtR1*, was amplified using primers with complementary overhangs. The amplified fragments were purified and assembled through application of NEBuilder HiFi (New England Biolabs, E2621).

Sequenced expression vectors were transformed into yeast strain INVSc1 (Invitrogen, C81000) and plated on uracil‐free selection media SD/–Ura for 3 d at 30°C. Positively transformed colonies were inoculated into 5 mL SD/–Ura medium and grown overnight at 30°C with shaking. After cultivation for 24 h, they were transferred to 50 mL SD/–Ura medium in a 250 mL flask and grown for another 24 h. To induce protein expression, pellet cells were resuspended in 50 mL SD/–Ura medium containing 2% galactose, supplemented with 5 mg/L riboflavin and 75 mg/L 5‐aminolevulinic acid (ALA). For feeding assays, purified substrates were added to a final concentration of 5 μM. After 72 h of cultivation, cultures were extracted with hexane, and the organic extract was then dried by rotary evaporation.

### Diterpenoid production via metabolic engineering

To obtain sufficient *syn*‐pimaradiene and *syn*‐pimaradien‐19,6β‐olide for feeding assays, the previously developed *E. coli* modular metabolic engineering system was employed (Cyr et al., 2007). For production of *syn*‐pimaradiene, a pDEST17/OsKSL4 construct was co‐transformed with a compatible pGG*s*C construct (expressing a GGPP synthase and *OsCPS4*) into the OverExpress C41 (DE3) strain (Lucigen, 60442‐1). For the production of *syn*‐pimaradien‐19,6β‐olide, the previously described pCDFDuet/AtR1/DEST::OsKSL4, pETDuet/CYP99A2/DEST::CYP76M8, and pCOLADuet/DEST::OsMS2 were co‐transformed into C41 cells pretransformed with pGG*s*C. In both cases, the resulting recombinant bacteria were cultured in 50 mL NZY liquid medium containing the appropriate antibiotics at 37°C overnight. These cultures were then used to inoculate 500 mL TB liquid medium with relevant antibiotics and shaken at 37°C until the OD_600_ reached 0.6–0.8. The cultures were transferred to a 16°C incubator shaker, supplemented with 75 mg/L ALA and 5 mg/L riboflavin, and then induced with 1 mM Isopropyl β‐D‐1‐thiogalactopyranoside (IPTG) after 1 h. In total, 3 × 1 L batches of the relevant culture were grown. After being cultured for 72 h, the resulting diterpenoids were extracted with an equal volume of 10% (v/v) ethyl acetate/hexane, which were separated and then dried via rotary evaporation under vacuum.

### Diterpenoid isolation and purification

The separated organic extracts from feeding assays or metabolic engineering cultures were fractionated over a 4 g silica column on a Grace Reveleris flash chromatography system with UV detection and automated injector and fraction collector, at a flow rate of 15 mL/min. Briefly, the column was pre‐equilibrated with hexanes before sample injection, followed by 100% hexane (0–4 min), 0%–100% acetone (4–5 min), 100% acetone (5–8 min), with peak‐based fraction collection (15 mL maximum per tube). As elucidated by GC–MS analysis, the olefin product eluted in the hexane fraction while the alcohol product was found in the acetone fraction. These fractions were dried under N_2_ and resuspended in 2 mL of a 1:9 water to methanol solution and filtered through a 0.2 μm filter (Thermo Scientific, F2513‐16).

Fractions were further separated using an Agilent 1200 series HPLC instrument equipped with a diode array UV detector and automated injector and fraction collector, over a semi‐preparative C18 column (Agilent InfinityLab Poroshell 120 EC‐C18, 4.6 × 150 mm, 4 μm, 693970‐902) run at 0.5 mL/min. The column was preequilibrated with 50% (v/v) acetonitrile/water, and the sample was injected, followed by washing (0–2 min) with same acetonitrile/water mix, then the percentage of acetonitrile increased to 100% (2–22 min), and final elution with 50% (v/v) acetonitrile/water (22–25 min), with time‐based fraction collection. Fractions containing pure compounds were dried under N_2_.

### GC–MS analyses

GC–MS analyses were performed using an Agilent 8890 GC System coupled with a 5977B quadrupole mass spectrometer in electron ionization (70 eV) mode using an HP‐5 MS column (Agilent, 19091S‐433) with helium as the carrier gas (1 mL/min). Samples (1 μL) were introduced in a splitless mode via a 7650 A automatic liquid sampler by injection at 250°C. The following temperature program was used: Initial column oven temperature set to 50°C, maintained for 3 min, followed by a ramp rate of 15°C/min to 300°C, which was held for 3 min. Mass ion values were recorded in the range from 60 to 600, with detection beginning 13 min after sample injection until the end of the run.

### NMR analysis

The purified compound was dissolved in 0.5 mL deuterated chloroform (CDCl_3_). NMR spectra were acquired on a Bruker AVIII‐800 spectrometer equipped with a 5 mm HCN cryogenic probe, using TopSpin v3.2 software. Analysis was carried out at 25°C. Chemical shifts were calculated by reference to those known for CDCl_3_ signals (^13^C 77.23 ppm, ^1^H 7.24 ppm) offset from tetramethylsilane (TMS). All spectra were acquired using standard programs from the TopSpin v3.2 software, with collection of 1D ^1^H NMR and 2D double‐quantum filtered correlation spectroscopy (DQF‐COSY), heteronuclear single‐quantum coherence (HSQC), heteronuclear multiple‐bond correlation (HMBC), HMQC‐COSY, and NOESY (800 MHz), as well as 1D ^13^C NMR (201 MHz) spectra.

### Creation of transgenic plants

The CRISPR/Cas9–2gRNA plasmids were constructed as previously described (Zhou et al., 2014). Briefly, guide RNA (gRNA) was assembled from two oligonucleotides for each target (Table S3), and then inserted into the *Btg*ZI and *Bsa*I cloning sites of the intermediate vector *pENTR‐gRNA* in sequence. The resulting vectors were then cloned into the Gateway binary vector *pBY02‐Cas9* by LR reaction.

The overexpression constructs were produced by amplifying the coding sequences of *KOL4* and *KOL5* using primers containing the relevant restriction sites—that is, *KOL4*‐F‐*Kpn*I/R‐*Bam*HI and *KOL5*‐F‐*Eco*RI/R‐*Bam*HI, respectively. *KOL4* was then inserted into the *Kpn*I and *Bam*HI sites, while *KOL5* was inserted into the *Eco*RI and *Bam*HI sites of the destination vector *pBY02‐mUbi* using T4 DNA ligase (New England Biolabs, M0202).

The CRISPR and overexpression constructs were transformed into immature embryo‐derived calli of cv. Kitaake, and the CRISPR construct targeting *KOL4* into the *Δc4bgc* line, using *Agrobacterium tumefaciens* strain EHA105 as previously described (Hiei and Komari, 2008). Transformed calli and regenerated plantlets were selected on 1/2MS medium supplemented with hygromycin B (50 μg/mL).

### Genotyping

Genomic DNA was extracted from leaves of rice transgenic plants using Plant DNAzol Reagent (Invitrogen, 10978021). Target DNA fragments were amplified via PCR with gene‐specific primers (Table S3), and their lengths were assessed by agarose gel electrophoresis. The PCR reactions were treated with ExoSAP‐IT (Applied Biosystems, 78201.1.ML) before being sent for Sanger sequencing.

Knock‐out lines were generated by screening T0 plants, with heterozygous plants further screened in the corresponding T1 and T2 generations to isolate homozygous lines. Simultaneously, the presence of the Cas9 construct was also assayed by PCR analysis for selection of the final Cas9‐free homozygous lines.

Overexpression T0 lines were also screened by PCR. Positive transformants were grown for several generations on media containing hygromycin B (50 μg/mL) as needed to acquire non‐segregating homozygous lines.

### RNA isolation and RT‐PCR

Total RNA was isolated from 7‐d‐old rice seedlings using a plant RNA isolation kit (Qiagen, 74904) according to the manufacturer's instructions. The RNA sample (3 mg) was used for first‐strand cDNA synthesis with RevertAid reverse transcriptase (Thermo Scientific, K1622). RT‐PCR was performed with Taq Master Mix (Promega, M7832) using *OsActin1* (LOC_Os03g61970) as a control. The primers used for RT‐PCR are listed in Table S3.

### Phytochemical analyses

A previously developed liquid chromatography with tandem mass spectrometry (LC–MS/MS) method has been successfully applied to detect LRD metabolism using copper chloride (CuCl_2_)‐induced leaves and root exudates (Lu et al., 2018; Zhang et al., 2021). Briefly, leaves of 3‐week old seedlings were cut into 5 cm pieces, and 0.1 g was induced by floating on a solution of 0.5 mM CuCl_2_. After 72 h, these leaves were frozen in liquid Nitrogen and ground into a fine powder. Extractions were carried out by shaking in 3 mL methanol for 72 h in the cold room (4°C). For each sample, 4.62 μg sclareol was added as an internal standard. The extracts were subjected to filtration (Thermo Scientific, F2513‐2) using a glass syringe, dried under N_2_, and resuspended in 1 mL methanol.

LC–MS/MS analyses were performed with 15 μL sample injections over an Ascentis C18 column (Supelco, 581301‐U) using an Agilent Technologies 1100 Series HPLC system coupled to both a UV‐Vis diode array detector and an Agilent Technologies Mass Selective Trap SL detector located in the W. M. Keck Metabolomics Research Laboratory of Iowa State University. A binary gradient consisting of water with 0.1% (v/v) formic acid (buffer A) and acetonitrile with 0.1% (v/v) formic acid (buffer B) was used. The solvent gradient elution program was as follows: initial stage, 40% buffer A; 0–13 min, a linear gradient from 40% buffer B to 85% buffer B; 13–14 min, a linear gradient from 85% buffer B to 100% buffer B; 14–14.5 min, a linear gradient from 100% buffer B to 40% buffer B. The known rice LRD phytoalexins were identified based on the calculated monoisotopic mass of the molecular ion for each [M + H]^+^ with an isolation selection window of ± 0.5 *m/z*. The isolated masses were re‐ionized and the resulting mass spectra recorded.

The isolated parent ions and quantified secondary ions, together with corresponding retention time (RT), based on optimization with the available authentic standards, for the known rice LRD phytoalexins were as follows: momilactone A, *m/z* 315.2 → 271.2, RT = 11.1 min; momilactone B, *m/z* 331.2 → 269.2, RT = 9.0 min; *epi*‐momilactone C, *m/z* 317.2 → 271.2, RT = 8.2 min; phytocassane A, *m/z* 317.2 → 299.2, RT = 7.6 min; phytocassane B, *m/z* 335.2 → 317.2, RT = 7.3 min; phytocassane C, *m/z* 319.2 → 301.2, RT = 6.8 min; phytocassane D, *m/z* 317.2 → 299.2, RT = 9.7 min; phytocassane E, *m/z* 317.2 → 299.2, RT = 8.3 min; phytocassane F, *m/z* 333.2 → 315.2, RT = 7.3 min; oryzalexin A, *m/z* 303.2 → 285.2, RT = 10.0 min; oryzalexin B, *m/z* 303.3 → 286.2, RT = 9.7 min; oryzalexin C, *m/z* 301.2 → 283.2, RT = 9.6 min; oryzalexin D, *m/z* 287.2 → 269.2 (147.2), RT = 9.6 min; oryzalexin E, *m/z* 287.2 → 269.2, RT = 11.3 min; oryzalexin S, *m/z* 287.2 → 269.2 (241.2), RT = 9.7 min. For the internal standard sclareol, *m/z* 273.2 → 163.1, RT = 13.5 min. The relative amount of each LRD was determined with the DataAnalysis software of Agilent 6300 Ion Trap LC/MS Systems, by comparison of total ion peak areas to that of sclareol.

### Fungal infection assays

*Magnaporthe oryzae* strain O254 was used, and infection assays were performed as previously described (Sesma and Osbourn, 2004). Briefly, seedlings at the third leaf stage were inoculated by spraying. Three pots of nine plants were used per line and per experiment. Each pot was sprayed with 3 mL of a suspension of 10^5^ conidia per mL. The sprayed plants were further incubated in the growth chamber with setting conditions (85% relative humidity and a 14 h light/10 h dark photoperiod at 25°C) for 6 d to score disease symptoms. Leaf lesions were scanned and analyzed with Assess 2.0 software (American Phytopathological Society). Blast lesions were evaluated based on lesion number and lesion extension of disease symptoms as previously reported (Tucker et al., 2010).

### Bacterial infection assays

Bacterial infections were carried out by infiltration of the leaves of 3‐week‐old seedlings. *Xoo* strain PXO99^A^ or *Xoc* strain BLS256 were grown on TSA plates (1% Tryptone, 1% sucrose, 0.1% glutamic acid, and 1.5% agar, pH 6.6 to 6.8) at 28°C for 2–3 d, then scraped off and resuspended in sterile de‐ionized water. This suspension was then adjusted to an optical density of 0.5 at 600 nm, and infiltrated into leaves with a needleless syringe, with five spots inoculated on each leaf. After 3 d, leaf fragments containing the inoculated spots were harvested and ground in a sterile mortar with the addition of 2 mL sterile water. The subsequent solutions were serially diluted and spread on TSA plates to count colonies for conversion into colony‐forming units per infiltration spot. This analysis was conducted three times in parallel for each rice line.

### DAB staining

3,3′‐diaminobenzidine (DAB) (1 mg/mL) was prepared as previously described (Daudi and O'Brien, 2012). For the detection of the accumulation of hydrogen peroxide (H_2_O_2_) in rice leaves showing the lesion‐mimicking symptom, DAB staining was performed. In brief, 8‐week‐old rice leaves were sampled and stained in the DAB solution for at least 8‐h in the dark. The stained leaves were later boiled in the 95% ethanol for 10 min and bleached out the chlorophyll using the 95% ethanol for 2 d.

### Bioinformatic analysis

Gene annotation information for the *OsKO*(*L*)*s* from *O. sativa* ssp. japonica cv. Nipponbare (Table S1) was obtained from RGAP (http://rice.uga.edu) and RAP‐DB (http://rapdb.dna.affrc.go.jp). Presence/Absence variation of *KOL4* and *KOL5* was assessed by BLAST searches of the Gramene *Oryza* database, with the identified genes then downloaded and used for sequence alignment. The conservation of these genes was more broadly determined by examination of the relevant loci at RiceVarMap2 (Zhao et al., 2021).

### Oryza datasets

*O. sativa*, *O. rufipogon*, *O. nivara*, *O. barthii*, *O. glaberrima*, *O. glumaepatula*, *O. meridionalis*, *O. punctata*, *O. brachyantha*, and *L. perrieri* protein sequences and gene annotations were downloaded from EnsemblPlants (http://plants.ensembl.org). *O. officinalis* protein sequences and gene annotations from PRJCA029552 (https://osf.io/y4gb5). *O. australiensis*, *O. coarctata*, and *O. meyeriana* genome assemblies from NCBI (PRJNA591699, PRJNA439330, PRJNA1039468).

### Phylogenetic analysis

A total of 310 single‐copy syntenic genes found in the 23 *Oryza* (sub) genomes and the outgroup genome were used for the phylogenomic analysis. Syntenic homologous genes were aligned with MAFFT, searched with BLASTP, and trimmed with Gblocks. Alignment files were subjected to IQ‐TREE with default settings (1,000 bootstrap iterations). Divergence times were generated using a molecular calibration of 15 million years for the crown age of *Oryza*.

### Synteny analysis

Genomes and protein sequences of *Oryza* species used in phylogenetic analysis were loaded to MCscan, a package from JCVI (Python version). Synteny comparison was performed using LAST. The syntenic blocks were constructed and visualized with default settings.

## CONFLICTS OF INTEREST

The authors declare no conflicts of interest.

## AUTHOR CONTRIBUTIONS

Z.W. helped design and conduct most experiments, analyzed the data, and drafted the manuscript. Y.F. conducted infection assays. L.Y. performed phylogenomic analyses. M.X. helped with infection assays and chemotyping. K.H. performed structural analysis. R.L. and S.N.C. performed rice genetic transformation. B.Y. helped design the experiments and supervise the rice genetic transformations. R.J.P. designed the experiments, supervised the study, and revised the manuscript. All authors have read and approved the contents of this paper.

## Supporting information

Additional Supporting Information may be found online in the supporting information tab for this article: http://onlinelibrary.wiley.com/doi/10.1111/jipb.70262/suppinfo

**Figure S1.** Catalytic activity of *OsKOL4* and *OsKOL5*
**Figure S2.** NMR structural analysis of 2α‐hydroxy‐*syn*‐pimaradien‐19,6β‐olide (*epi*‐momilactone C)
**Figure S3.** ^1^H NMR spectrum of 2α‐hydroxy‐*syn*‐pimaradien‐19,6β‐olide (*epi*‐momilactone C) in CDCl_3_
**Figure S4.** ^13^C NMR spectrum of 2α‐hydroxy‐*syn*‐pimaradien‐19,6β‐olide (*epi*‐momilactone C) in CDCl_3_
**Figure S5.** Targeted mutagenesis of *OsKOL4* or *OsKOL5*
**Figure S6.** Targeted simultaneous mutagenesis of both *OsKOL4* and *OsKOL5*
**Figure S7.** Expression analysis of over‐expression (OE) transgenic rice plants
**Figure S8.** *OsKOL4* affects plant height in rice
**Figure S9.** Effect of genetic manipulation of *OsKOL4* and *OsKOL5* on production of momilactone B
**Figure S10.** Effect of genetic manipulation of *OsKOL4* and *OsKOL5* on production of *epi*‐momilactone C
**Figure S11.** Susceptibility of *OsKOL4* and *OsKOL5* knockout and overexpression plants to *X. oryzae* pv. *oryzicola* (*Xoc*)
**Figure S12.** Lesion mimic phenotype associated with *kol4*
**Figure S13.** Targeted mutagenesis of *OsKOL4* in *Δc4bgc*
**Figure S14.** Phylogenetic and syntenic analyses of KO(L) homologs in *Oryza* AA genome type species
**Figure S15.** KOL variation in rice
**Table S1.** List of *KO*(*L*) genes in *Oryza sativa*
**Table S2.** ^1^H and ^13^C NMR spectral data for 2α‐hydroxy‐*syn*‐pimaradien‐19,6β‐olide (*epi*‐momilactone C) in CDCl_3_
**Table S3.** List of primers used in this study
