# Supplementary material for: Divergent roles of ent‐kaurene oxidase paralogs in rice momilactone biosynthesis
Source: J Integr Plant Biol. 2026 Apr 21;68(10):3776–89. doi: 10.1111/jipb.70262 (PMC13629296; doi:10.1111/jipb.70262)
Supplement: Supplementary file 1 [file JIPB-68-3776-s001.docx]

**Supplemental Data for:**

**Divergent roles of *ent*-kaurene oxidase paralogs in rice momilactone biosynthesis**

**Zhibiao Wang^1,2^, Yiling Feng^2^, Leiming You^1^, Meimei Xu^2^, Kristin Helwig^2^, Riqing Li^3^, Si Nian Char^3^, Bing Yang^3,4^, and Reuben J. Peters^2*^**

^1^School of Life Sciences, Beijing University of Chinese Medicine, Beijing 100029, China.

^2^Roy J. Carver Department of Biochemistry, Biophysics & Molecular Biology, Iowa State University, Ames, IA 50011, USA.

^3^Division of Plant Sciences, Bond Life Sciences Center, University of Missouri, Columbia, MO 65211, USA.

^4^Donald Danforth Plant Science Center, St. Louis, MO 63132, USA.

**^*^**Correspondence: Reuben J. Peters [(rjpeters@iastate.edu)](mailto:(rjpeters@iastate.edu))

**Table S1. List of *KO(L)* genes in *Oryza sativa*.**

| **Gene** | **Protein** | **RGAP** | **RAP-DB** |
| --- | --- | --- | --- |
| *OsKO1.1* | CYP701A7 |  | *Os06g0570600* |
| *OsKO1.2* |  |  | *Os06g0570566* |
| *OsKO2* | CYP701A6 | *LOC_Os06g37364* | *Os06g0570100* |
| *OsKO3* | CYP701A19 | *LOC_Os06g37330* | *Os06g0569900* |
| *OsKOL4* | CYP701A8 | *LOC_Os06g37300* | *Os06g0569500* |
| *OsKOL5* | CYP701A9 | *LOC_Os06g37224* | *Os06g0568600* |

**Table S2. ^1^H and ^13^C NMR spectral data for 2α-hydroxy-*syn*-pimaradien-19,6β-olide (*epi*-momilactone C) in CDCl_3_.**

| **Position** | **δH** | **δC** |
| --- | --- | --- |
| 1a | 1.25 (1H, m) | 42.2 |
| 1b | 1.62 (1H, d, *J* = 4.8 Hz) |  |
| 2 | 4.16 (1H, tt, *J* = 10.3, 5.8 Hz) | 64.78 |
| 3a | 2.70 (1H, dd, *J* = 14.6, 6.4 Hz) | 39.46 |
| 3b | 1.3 (1H, m) |  |
| 4 |  | 43.77 |
| 5 | 1.88 (1H, d, *J* = 4.4 Hz) | 45.29 |
| 6 | 4.88 (1H, t, *J* = 9.76, 4.88 Hz) | 73.42 |
| 7 | 5.65 (1H, d, *J* = 5.2 Hz) | 114.72 |
| 8 |  | 148.29 |
| 9 | 1.63 (1H, d, *J* = 4.8 Hz) | 51.28 |
| 10 |  | 34.25 |
| 11a | 1.26 (1H, m) | 23.28 |
| 11b | 1.67 (1H, dq, *J* =12.0, 3.8 Hz) |  |
| 12 | 1.54 (2H, m) | 37.09 |
| 13 |  | 40.04 |
| 14a | 2.14 (1H, d, *J* = 12.0 Hz) | 47.39 |
| 14b | 1.99 (1H, dd, *J* = 12.0, 2.2 Hz) |  |
| 15 | 5.82 (1H, dd, *J* = 17.4, 10.7 Hz) | 149.13 |
| 16a | 4.95 (1H, d, *J* = 17.4 Hz) | 110.02 |
| 16b | 4.91 (1H, d, *J* = 10.72 Hz) |  |
| 17 | 0.85 (3H, s) | 21.96 |
| 18 | 3.47 (3H, s) | 50.91 |
| 19 |  | 181.43 |
| 20 | 0.94 (3H, s) | 24.03 |

**Table S3. List of primers used in this study**

| **Primer Name** | **Primers sequences** |
| --- | --- |
| **Primers for constructs in yeast** | |
| CYC1ter-F | GCTTATCATGTAATTAGTTATGTCACG |
| AtR1-KOL4-R | GGCTACGAGCATCGACTCCATtttttttttttttatgatgttaaggtatag |
| KOL4-F | ATGGAGTCGATGCTCGTAGCC |
| KOL4-R | cgtgacataactaattacatgataagcTCACATCCTTCCTCTGGGCTTG |
| AtR1-KOL5-R | GGCTGCGAGCAGCGACTCCATtttttttttttttatgatgttaaggtatag |
| KOL5-F | ATGGAGTCGCTGCTCGCAGCC |
| KOL5-R | cgtgacataactaattacatgataagcCTAGCTTCCTCTGGGCTTGAG |
| **Primers for constructs *in planta*** | |
| gKOL4-1F | tgttGGCCGTCGGGGGCCTCGTCG |
| gKOL4-1R | aaacCGACGAGGCCCCCGACGGCC |
| gKOL4-2F | gtgtGCTCGCCGACAAGCTCGTCG |
| gKOL4-2R | aaacCGACGAGCTTGTCGGCGAGC |
| gKOL5-1F | tgttGGGAGGGATCGGCGTGGCTG |
| gKOL5-1R | aaacCAGCCACGCCGATCCCTCCC |
| gKOL5-2F | gtgtGTGGTGCCGCCCAAGGACCG |
| gKOL5-2R | aaacCGGTCCTTGGGCGGCACCAC |
| gKOL4/5-1F | tgttTGAAGTTCAGAGGGGAATGT |
| gKOL4/5-1R | aaacACATTCCCCTCTGAACTTCA |
| gKOL4/5-2F | gtgtGTTTGGGAGGGAGATATCCA |
| gKOL4/5-2R | aaacTGGATATCTCCCTCCCAAAC |
| KOL4-F-KpnI | cggggtaccATGGAGTCGATGCTCGTAGCC |
| KOL4-R-BamHI | cgcggatccTCACATCCTTCCTCTGGGCTTG |
| KOL5-F-EcoRI | atttcttgcagatccgaattcATGGAGTCGCTGCTCGCAGCC |
| KOL5-R-BamHI | cttccatgggtcgacggatccCTAGCTTCCTCTGGGCTTGA |
| **Primers for transgenic plants identification** | |
| KOL4-1F | GGGAGCGAAACCTCTCTTTC |
| KOL4-1R | TGCGACACCAGAAATTACCA |
| KOL4-2F | CTACCAAAAGATGGCGAAGC |
| KOL4-2R | GTGCAATCCTCTCCTTCTGC |
| KOL5-1F | CCCACACACTTGCTCCTTCT |
| KOL5-1R | AACCAACCACAACCTTCTCC |
| KOL5-2F | TGCGCAGGTACAAATCTCAA |
| KOL5-2R | CCTCTTGATCAACGCGTTCA |
| OsCas9-F | GGGTAATGAACTCGCTCTGC |
| OsCas9-R | TGGCGTCAAGAACTTCCTTTG |
| Hyg-F | CCGCTCGTCTGGCTAAGATC |
| Hyg-R | GTCCTGCGGGTAAATAGCTG |
| **Primers for RT-PCR** | |
| KOL4-RT-F | CGTTTGCAAAATGGTCTGAA |
| KOL4-RT-R | GGGAATGTGGGTCAAGAGAA |
| KOL5-RT-F | CCTGGTTTACCCGTTATTGG |
| KOL5-RT-R | GGGTCAACAGCAACCAATTT |
| OsActin1-F | CTCAGCACATTCCAGCAGAT |
| OsActin1-R | ACAGATAGGCCGGTTGAAAA |

**Figure S1. Catalytic activity of OsKOL4 and OsKOL5.**

**(A)** GC-MS chromatogram from a feeding assay of OsKOL4 with *syn*-pimaradiene (1), with the production of *syn*-pimaradien-3β-ol (2).

**(B)** Mass spectrum of 2.

**(C)** GC-MS chromatogram from a feeding assay of OsKOL5 with *syn*-pimaradiene (1), with the production of a novel product (3).

**(D)** Mass spectrum of 3.

**(E)** Reactions catalyzed by OsKOL4 and OsKOL5. OsKOL4 catalyzed 3β hydroxylation of 1, while OsKOL5 converts 1 to a novel hydroxylated product.

**Figure S2. NMR structural analysis of 2α-hydroxy-*syn*-pimaradien-19,6β-olide (*epi*-momilactone C).**

**(A)** Structure of 2α-hydroxy-*syn*-pimaradien-19,6β-olide (*epi*-momilactone C) with carbon numbering.

**(B)** COSY, HMBC and NOESY correlations used to assign configurations.

**Figure S3. ^1^H NMR spectrum of 2α-hydroxy-*syn*-pimaradien-19,6β-olide (*epi*-momilactone C) in CDCl_3_.**

**Figure S4. ^13^C NMR spectrum of 2α-hydroxy-*syn*-pimaradien-19,6β-olide (*epi*-momilactone C) in CDCl_3_.**

**Figure S5. Targeted mutagenesis of *OsKOL4* or *OsKOL5*.**

**(A)** Upper: Gene structure of *OsKOL4* and the location of two target gRNAs.

The start codon (ATG) and the stop codon (TGA) are indicated. Black boxes indicate the exon, grey boxes show the 5’ and 3’-untranslated regions, and lines between boxes represent the intron. The location of target sites are indicated by red lines.

Lower: Detection of targeted *OsKOL4* mutations based on sequencing.

The WT sequence appears at the top, with the PAM sequence shown in green and the target sequences highlighted in red. Nucleotide changes in each line are listed. DNA insertions, point mutations are shown as blue letters. Deletions are shown as blue dashes. +, insertion. −, deletion.

**(B)** Upper: Gene structure of *OsKOL5* and the location of two target gRNAs.

The start codon (ATG) and the stop codon (TAG) are indicated. Black boxes indicate the exon, grey boxes show the 5’ and 3’-untranslated regions, and lines between boxes represent the intron. The location of target sites are indicated by red lines.

Lower: Detection of targeted *OsKOL5* mutations based on sequencing.

The WT sequence appears at the top, with the PAM sequence shown in green and the target sequences highlighted in red. Nucleotide changes in each line are listed. Deletions are shown as blue dashes. −, deletion.

**Figure S6. Targeted simultaneous mutagenesis of both *OsKOL4* and *OsKOL5*.**

**(A)** Upper: Gene structure of *OsKOL4* and the location of two target gRNAs.

The start codon (ATG) and the stop codon (TGA) are indicated. Black boxes indicate the exon, grey boxes show the 5’ and 3’-untranslated regions, and lines between boxes represent the intron. The location of target sites are indicated by red lines.

Lower: Detection of targeted *OsKOL4* mutations based on sequencing.

The WT sequence appears at the top, with the PAM sequence shown in green and the target sequences highlighted in red. Nucleotide changes in each line are listed. DNA insertions are shown as blue letters. Deletions are shown as blue dashes. +, insertion. −, deletion.

**(B)** Upper: Gene structure of *OsKOL5* and the location of two target gRNAs.

The start codon (ATG) and the stop codon (TAG) are indicated. Black boxes indicate the exon, grey boxes show the 5’ and 3’-untranslated regions, and lines between boxes represent the intron. The location of target sites are indicated by red lines.

Lower: Detection of targeted *OsKOL5* mutations based on sequencing.

The WT sequence appears at the top, with the PAM sequence shown in green and the target sequences highlighted in red. Nucleotide changes in each line are listed. DNA insertions are shown as blue letters. Deletions are shown as blue dashes. +, insertion. −, deletion.

**Figure S7. Expression analysis of over-expression (OE) transgenic rice plants.**

**(A)** Semi-quantitative RT-PCR of *OsKOL4* expression in wild-type and 7-day-old *KOL4-OE* rice seedlings.

*OsActin1* was used as an internal control.

**(B)** Semi-quantitative RT-PCR of *OsKOL5* expression in wild-type and 7-day-old *KOL5-OE* rice seedlings.

*OsActin1* was used as an internal control.

**Figure S8. *OsKOL4* affects plant height in rice.**

**(A)** Plant phenotypic comparison between WT, *OsKOL4* knockout lines, *OsKOL5* knockout lines, *OsKOL4/5* double knockout lines, *OsKOL4-OE* lines, and *OsKOL5-OE* lines at the heading stage. Scale bar = 10 cm.

**(B)** Average plant height of WT, *OsKOL4* knockout lines, *OsKOL5* knockout lines, *OsKOL4/5* double knockout lines, *OsKOL4-OE* lines, *OsKOL5-OE* lines at the heading stage (*n* = 6). Values are given as means ± *SE*. ***P* < 0.01; ****P* < 0.001 compared with the wild-type (Student’s *t*-test).

**Figure S9. Effect of genetic manipulation of *OsKOL4* and *OsKOL5* on production of momilactone B.**

**(A)** Effect from individual (left) or double knock-outs (right) (as indicated).

**(B)** Effect from over-expression (as indicated).

**Supplemental Figure S10. Effect of genetic manipulation of *OsKOL4* and *OsKOL5* on production of *epi*-momilactone C.**

**(A)** Effect from individual (left) or double knock-outs (right) (as indicated).

**(B)** Effect from over-expression (as indicated).

**Figure S11. Susceptibility of *OsKOL4* and *OsKOL5* knockout and overexpression plants to *X. oryzae pv. oryzicola* (*Xoc*).**

**(A)** *OsKOL4* and *OsKOL5* loss-of-function mutants exhibit increased susceptibility to *Xoc*. Leaves from 3-week-old plants of WT, *kol4*, *kol5* and *kol4/5* lines after inoculation with *Xoc* strain BLS256 via needless syringe infiltration.

**(B)** Bacterial number per inoculation spot of WT, *kol4*, *kol5* and *kol4/5* lines 3 days post infection with *Xoc* strain BLS256.

**(C)** *OsKOL4* and *OsKOL5* overexpression lines exhibit enhanced resistance to *Xoc*. Leaves from 3-week-old plants of WT, *KOL4-OE* and *KOL5-OE* lines after inoculation with *Xoc* strain BLS256 via needless syringe infiltration.

**(D)** Bacterial number per inoculation spot of WT, *KOL4-OE* and *KOL5-OE* lines 3 days post infection with *Xoc* strain BLS256.

Values in **(C**, **D)** are given as means ± *SE*. Significant differences are indicated by different letters (*P* < 0.05, two-sided Fisher’s least significant difference).

**Figure S12. Lesion mimic phenotype associated with *kol4*.**

Leaves from the heading stage of plants grown in a common growth chamber. WT and *kol5* are healthy, while *kol4* and *kol4/5* exhibit mild to severe lesion mimic phenotype.

**(A)** Representative WT.

**(B)** Range of phenotypes observed with *kol4-6* line.

**(C)** Representative *kol5-6*

**(D)** Range of phenotypes observed with *kol4/5-3* line.

Δ**c4bgc*-*OsKOL4 (WT with** Δ**c4bgc)**

GCGGGCGCGGCGGCGGTGGCGGCCGTCGGGGGCCTCGTCGCGGCGGCCGCGCTCGCCGACAAGCTCGTCGCGGCGCCGCCGCCGCGCAAGAACCGCGCCAACCCGCCTCCAGgtaattaattaatccagctcgccgtgt

Δ**c4bgc-CR_KOL4 #1 (biallelic mutations)**

Allele 1: (−33 bp)

GCGGGCGCGGCGGCGGTGGCGGCCGTCGGGGGCCTCG~~TCGCGGCGGCCGCGCTCGCCGACAAGCTCGTCG~~CGGCGCCGCCGCCGCGCAAGAACCGCGCCAACCCGCCTCCAGgtaattaattaatccagctcgccgtgt

Allele 2: (+1 bp)

GCGGGCGCGGCGGCGGTGGCGGCCGTCGGGGGCCTCG**A**TCGCGGCGGCCGCGCTCGCCGACAAGCTCGTCGCGGCGCCGCCGCCGCGCAAGAACCGCGCCAACCCGCCTCCAGgtaattaattaatccagctcgccgtgt

Δ**c4bgc-CR_KOL4 #3**

Allele 1: (homozygous for composite base change: −38 bp & +28 bp)

GCGGGCGCGGCGGCGGTGGC~~GGCCGTCGGGGGCCTCGTCGCGGCGGCCGCGCTCGCCG~~**AAGAACATATAAGTTCGTCGCGGCGGTG**ACAAGCTCGTCGCGGCGCCGCCGCCGCGCAAGAACCGCGCCAACCCGCCTCCAGgtaattaattaatccagctcgccgtgt

Allele 2: (homozygous for composite base change: −38 bp & +28 bp)

GCGGGCGCGGCGGCGGTGGC~~GGCCGTCGGGGGCCTCGTCGCGGCGGCCGCGCTCGCCG~~**AAGAACATATAAGTTCGTCGCGGCGGTG**ACAAGCTCGTCGCGGCGCCGCCGCCGCGCAAGAACCGCGCCAACCCGCCTCCAGgtaattaattaatccagctcgccgtgt

Δ**c4bgc-CR_KOL4 #5:**

Allele 1: (−1 bp)

GCGGGCGCGGCGGCGGTGGCGGCCGTCGGGGGCCTC~~G~~TCGCGGCGGCCGCGCTCGCCGACAAGCTCGTCGCGGCGCCGCCGCCGCGCAAGAACCGCGCCAACCCGCCTCCAGgtaattaattaatccagctcgccgtgt

Allele 2: (−32 bp)

GCGGGCGCGGCGGCGGTGGCGGCCGTCGGGGGCCTC~~G~~TCG~~CGGCGGCCGCGCTCGCCGACAAGCTCGTCGCG~~GCGCCGCCGCCGCGCAAGAACCGCGCCAACCCGCCTCCAGgtaattaattaatccagctcgccgtgt

**Figure 13. Targeted mutagenesis of *OsKOL4* in** *Δ****c4bgc*.**

Partial sequences showing the CRISPR-induced insertions and deletions in edited lines. Strikethrough letters indicate deletions, and bold light blue letters are insertions. Green letters are PAM sequences for CRISPR-Cas9 targets. Lower letters are intron sequences.

**Figure S14. Phylogenetic and syntenic analyses of KO(L) homologs in *Oryza* AA genome type species.**

**(A)** Time-dated phylogenetic tree of 7 AA genome type *Oryza* species (*O. sativa*, *O. rufipogon*, *O. nivara*, *O. barthii*, *O. glaberrima*, *O. glumaepatula* and *O. meridionalis*). IQ-TREE was used to construct a maximum likelihood phylogeny using 310 single-copy genes. Divergence time was obtained using the molecular calibration for the crown age of *Oryza* (15 million years). MYA, million years ago.

**(B)** Syntenic analysis of the KO gene tandem array of 7 AA genome type *Oryza* species. The blue, green, cyan, red and yellow ribbons show the syntenic relationships between KO1, KO2, KO3, KOL4 and KOL5 respectively.

**(C)** Phylogenetic tree of KO(L) homologs in 7 AA genome type *Oryza* species. IQ-TREE was built using the maximum likelihood method with 1,000 bootstrap values.

**Figure S15. *KOL* variation in rice.**

From RiceVarMap2.

**(A)** *KOL4*.

**(B)** *KOL5*.
